# Dr. Paul Carpenter

**Published:** 1918-08

**Authors:** 


**Dr. Paul Carpenter** of Pittsford, N. Y. died June 4, age 52. He graduated from the Jefferson Medical College in 1867.

